# *Schistosoma mansoni* Infection and Associated Determinant Factors among School Children in Sanja Town, Northwest Ethiopia

**DOI:** 10.1155/2014/792536

**Published:** 2014-04-01

**Authors:** Ligabaw Worku, Demekech Damte, Mengistu Endris, Habtie Tesfa, Mulugeta Aemero

**Affiliations:** ^1^School of Biomedical and Laboratory Sciences, University of Gondar, 196 Gondar, Ethiopia; ^2^Department of Biology, University of Gondar, 196 Gondar, Ethiopia

## Abstract

*Background*. Intestinal schistosomiasis is one of the most widespread parasitic infections in tropical and subtropical countries. *Objective*. To determine the prevalence of *S. mansoni *infection and associated determinant factors among school children in Sanja Town, northwest Ethiopia. *Methods*. A cross-sectional study was conducted from February to March, 2013. 385 school children were selected using stratified proportionate systematic sampling technique. Pretested questionnaire was used to collect sociodemographic data and associated determinant factors. Stool samples were examinedusing formol-ether concentration and Kato-Katz technique. Data were entered and analyzed using SPSS 20.0 statistical software. Multivariate logistic regression was done for assessing associated risk factors and proportions for categorical variables were compared using chi-square test. *P* values less than 0.05 were taken as statistically significant. *Results*. The prevalence of *S. mansoni *infection was 89.9% (*n* = 346). The overall helminthic infection in this study was 96.6% (*n* = 372). Swimming in the river, washing clothes and utensil using river water, crossing the river with bare foot, and fishing activities showed significant association with the occurrence of *S. mansoni *infection. *Conclusion*. *Schistosoma mansoni* infection was high in the study area. Therefore, mass deworming at least twice a year and health education for community are needed.

## 1. Background

Schistosomiasis is one of the most widespread parasitic infections in developing countries [1]. An estimated 779 million people are at risk, with 240 million infected cases and more than 200,000 deaths occurring each year due to schistosomiasis worldwide [2, 3]. In Africa, over 90% of disease burden is found in sub-Saharan Africa [4], where* S. mansoni* and* S. haematobium* are the main causative species of schistosomiasis in the continent with an estimation of 54 and 112 million individuals infected, respectively, and the risk of infection for* S. mansoni* and* S. haematobium* was 393 and 436 million, respectively [5].

Schistosomiasis is also one of the most common parasitic diseases and is widespread in many parts of Ethiopia, usually at an altitude between 1,200 and 2,000 meters above sea level [6]. Many reports show that intestinal schistosomiasis caused by* S. mansoni* is widely distributed in the country than urinary schistosomiasis caused by* S. haematobium* [7–15]. The main determinants for the distribution, transmission, and spreading of both Schistosoma species (*S. mansoni* and* S. haematobium*) in Ethiopia include water temperature, absence or presence of snail intermediate host, population movement, and water impoundment for irrigation and power [9].

Schistosomiasis affects human host by slow damage of the host organs due to granuloma formation around the eggs in the tissues. This leads to the development of fibrosis and chronic inflammation in the liver and causes severe damage including bleeding, renal failures, and cancer [16].

The high prevalence of this infection is closely correlated to infested water bodies (pond, stream, river, and dam) contact during crossing with bare foot, swimming, washing of clothes and utensil, playing, fishing, and irrigation activity [17]. School-aged children are the most affected group due to high exposure to infested water bodies. Growth retardation and poor school performance are adverse effects of the disease besides clinical manifestation and its complication [18]. Although several studies have been conducted on the prevalence of* S. mansoni* and their risk factors in Ethiopia, there is still a lack of epidemiological information in some localities of northwest Ethiopia. Therefore, the aim of this study was to determine the prevalence of* S. mansoni* infection and associated determinant factors among school children of Sanja General Elementary School in Sanja Town, northwest Ethiopia.

## 2. Methods

### 2.1. Study Design and Period

A cross-sectional study was conducted from February 1 to March 30, 2013, at Sanja Town, northwest Ethiopia.

### 2.2. Study Area

Sanja Town is located 792 km far from the capital city Addis Ababa on the roadside to Gondar-Humera. Sanja has an altitude of 1800 m above sea level with annual rainfall ranging from 800 to 1800 mm and annual temperature ranging from 25°C to 42°C. There are two elementary schools, one junior and one high school. One health center gives service for the dwellers of the town and the surrounding areas. There is a river known as Sanja that flows throughout the year. Sanja General Elementary School is located on the west of the main road. A total of 2079 (872 male, 1207 female) students were enrolled in the school for 2012/13 academic year.

### 2.3. Study Population

The study populations were Sanja General Elementary School children who were enrolled in the school during the study period.

### 2.4. Sampling and Sample Size Determination

The sample size was determined using statistical formula (*n* = (*z* ^2^ *p* (1 − *p*)*d* ^2^) considering 95% confidence interval and 50% prevalence. Based on this assumption, 385 study participants were selected from 2079 students. To select the study subjects, the students were first stratified according to their educational level (Grade 1 to Grade 8). Allocation of student was proportional to the number of students in each grade. Finally, the school children were selected using systematic sampling using class roster as the sampling frame. Every fifth pupil was selected for the study.

### 2.5. Data Collection

#### 2.5.1. Sociodemography and Determinant Factors Assessments

Sociodemography and possible determinant factors were assessed with pretested and standardized questionnaire, which was translated into the local language, Amharic.

#### 2.5.2. Detection and Quantification of Intestinal Helminths

The school children were provided with small, clean, dried, and leak proof container and clean wooden applicator stick. Then, they were informed to bring sizable stool sample of their own. Then, the stool sample was processed and diagnosed microscopically using formol-ether concentration and Kato-Katz techniques. Double Kato-Katz was employed to a template delivering 41.7 mg of stool [19]. Eggs counted for* S. mansoni* and other common intestinal helminths were recorded and later converted into eggs per gram of stool (EPG), multiplying by a factor of 24. Infection intensity (light, moderate, and high) was classified according to the World Health Organization (WHO) criteria [20].

#### 2.5.3. Quality Control

All the necessary reagents, chemicals, and the performance of kits were checked by known positive and negative samples before processing and examination of samples of the study subjects. The data was checked for completeness and any incomplete or misfiled questionnaires were recorrected under supervision. The slides were examined by two microscopists independently under the middle (40x) objective of the microscope. Negative samples were reexamined on the same day at the same time by another laboratory technologist. The result of laboratory examination was recorded on well-prepared format carefully. Ten percent of the total slide was randomly selected and reexamined at the end by experienced laboratory technologist at the University of Gondar, who was blinded for the first examination results.

#### 2.5.4. Data Analysis and Interpretation

Data were entered and analyzed using SPSS 20.0 (SPSS Inc., Chicago, 2011) software. Descriptive statistics was used to give a clear picture of background variables. The frequency distribution of both dependent and independent variables was worked out. Multivariate logistic regression was done for assessing associated risk factors and proportions for categorical variables were compared using chi-square test. *P* values less than 0.05 were taken as statistically significant.

#### 2.5.5. Ethical Considerations

Ethical clearance was obtained from research and ethics review committee of School of Biomedical and Laboratory Sciences, University of Gondar. Before starting of the actual data collection, permission was obtained from school director. Additionally, after explaining the importance of the study, an informed written consent was obtained from study participant's parent/guardian. An assent was also taken from the school children. Those children who were positive for* S. mansoni* and other intestinal parasites were treated according to the national protocol.

## 3. Results

### 3.1. Sociodemographic Characteristics

A total of 385 school children were included in the study. Their mean age was 12.7 years (range: 6 to 15 years) with standard deviation of 2.3. Among these, 132 (34.3%) were males and 253 (65.7%) were females. Most of the school children 312/385 (81%) were between 11 and 15 years. Of the 385 school children, 288 (74.8%) were from Sanja Town while 97 (25.2%) were from the surrounding rural areas (Table 1).

### 3.2. Prevalence and Egg Intensity of* S. mansoni *Infection

Among the total 385 school children examined, 372 (96.6%) were positive for* S. mansoni* and/or one or more other intestinal helminths. The most prevalent parasitic infection was intestinal schistosomiasis (*S. mansoni*) 346 (89.9%), followed by Hookworm 143 (37.1%),* Ascaris lumbricoides* 62 (16.1%),* Hymenolepis nana* 31 (8.1%),* Taenia* species 6 (1.6%), and* Trichuris trichiura* 3 (0.8%). Of the positive cases, 66.7% were females and 33.3% were males (Table 2).

Regarding parasitic load, the highest number of egg count per gram of faeces (EPG) for* S. mansoni* was 1776, which was observed only in one student. The intensity of infection using the Kato-Katz method among the total parasite infected school children 126 (32.7), 181 (47), and 72 (18.7%) was heavy, moderate, and heavy, respectively, whereas the intensity of* S. mansoni* infection 71 (18.4), 181 (47), and 72 (18.7%) was heavy, moderate, and heavy, respectively (Table 3).

### 3.3. Determinants of* S. mansoni* Infection

Of the total 385 school children examined, 119/132 (90.2%) male and 227/253 (89.7%) female school children were positive for schistosomiasis. The distribution of* S. mansoni* infection among each age group showed that 90.4% of 6–10 years and 89.7% of the 11–15 years were infected. Prevalence of* S. mansoni* infection among school children who lived in rural and urban areas was 88.7% and 90.3%, respectively (Table 4).

Determinant factors assessment for* S. mansoni* infection in general showed that swimming in the river, frequent swimming habit, washing clothes and utensil using river water, crossing the river with bare foot, and fishing activities were associated with* S. mansoni* infection (*P* < 0.05) (Table 4).

In multivariate analysis, swimming in the river and frequent swimming habit were associated with* S. mansoni* infection (*P* < 0.05). Children who swam in the river had 5.12 times (CI: 3.47, 17.89) higher risk of being infected with* S. mansoni* than those who did not have a swimming habit. School children who had swimming frequency of three or more times per week were having 4.83 times (CI: 1.37, 18.81) higher odds of being infected with* S. mansoni* than those who did not (Table 4).

*Schistosoma mansoni* infection was also significantly associated with the habit of washing clothes and utensils. Children who wash clothes and utensils using river water had 5.54 (CI: 2.89, 13.76) and 4.91 (2.43, 12.37) times higher odds of being infected with* S. mansoni* than those who did not, respectively. Children who cross the river with bare foot had 3.95 times (CI: 2.13, 7.61) higher risk of being infected with* S. mansoni* than those who did not cross the river. In addition, those students who had the habit of fishing were three times (CI: 2.14, 8.71) more likely to acquire* S. mansoni* infection (Table 3). Age, sex, residence, grade of student, and maternal/guardian education status had no statistically significant association with* S. mansoni* infection (Table 4).

## 4. Discussion

The prevalence rate of 89.9%* S. mansoni* infection was reported for the first time from the present study among school children in Sanja town. This study showed a higher prevalence rate of* S. mansoni* infection compared with other surveys conducted among school children from different parts of Ethiopia which reported prevalence ranging from 0.8% to 63% [21–33] and in other countries such as 16.5% Kenya [34], 27.8% Uganda [35], 2.7% Rwanda [36], 12.1% Nigeria [37], and 14.4% Brazil [38]. The reason why the observed infection prevalence varies in this study from other findings might be due to (1) the difference in water contact behavior of the school children (frequency of contact-infested water), ecological distribution of intermediate host (snail), local endemicity of the parasite, and sample size; (2) the difference in altitude and the temperature, which is favorable for the development and survival of snail; (3) the difference of method employed for stool examination and the time of study; and (4) the variation in awareness regarding transmission and prevention of* S. mansoni* infection between the study participants in this and previous studies.

The intensity of* S. mansoni* infection in this study shows 18.4% light, 47% moderate, and 18.7% heavy among the total* S. mansoni* positive school children while no heavy or moderate 16 infection was observed for hookworm* A. lumbricoides*, and* T. trichiura* (Table 2), which is quite different from that reported from Azezo (light 67.8%, moderate 19.8%, and heavy 3.1%) [32]; this might be due to the variation of infection rate.

The sex distribution of* S. mansoni* in the present study did not show a significant variation, which was similar to that reported from Rwanda and Nigeria [36, 37]. However, it was inconsistent with a research finding reported from different parts of Ethiopia such as Zarima, Tseda, and Gorgora in which the prevalence was slightly higher in females [26, 30, 31], whereas the prevalence was higher in males reported from Adwa, Mekelle, Chiga, Amibera, and Tikur Wuha [21, 23, 28, 33]. This difference might be due to similarity in water contact behavior of males and females such as swimming, crossing water bodies, washing clothes and/or utensils, and fishing in the present study.

The prevalence of* S. mansoni* showed equal distribution of infection in different age groups (6–10 and 11–15 years). This is inconsistent with reports of many researchers in different localities of Ethiopia [21–23, 26, 28] and Uganda [35] which was higher in the oldest group. Similarly, equal distribution of infection was observed in terms of residence. This is contrary to other findings conducted in Gorgora [26] and Tigray was higher in rural areas [38] while higher in urban area of Tikur Wuha [23]. This might be due to equal tendency of infested water exposure of both sex and proximity of Sanja River and other water bodies to the town and surrounding areas.

The habit of frequent contact with cercariae infested water such as swimming in the river, washing clothes and utensils using river water, crossing the river with bare foot, and fishing activities showed a statistically significant association with prevalence of* S. mansoni* infection. This is similar to the previous findings reported from (Adwa, Azezo, Zarima, Mekelle, Gorgora, and Amibera) Ethiopia, Kenya, and Rwanda [21, 26, 28, 31–34, 36]. The prevalence of* S. mansoni* was higher in school children who had a habit of frequent swimming than who did not. This might be due to the presence of cercariae infected water body/ies in the surrounding of study areas.

## 5. Conclusion 

Ninety percent of the school children were infected with* S. mansoni*. Swimming in the river, washing clothes and utensils using river water, crossing the river with bare foot, and fishing activities were the determinant factors identified in this study. Therefore, this calls the concerned bodies to take measures on the transmission of* S. mansoni*. Health education should be given to increase the awareness of school children about the risk of infested water contact. Application of mass deworming should be also considered for the students once in a year.

## Figures and Tables

**Table 1:** Sociodemographic characteristics of school children in Sanja General Elementary School, Sanja, northwest Ethiopia, 2013.

Sociodemographic characteristics	Number	Percent
Sex		
Male	132	34.3
Female	253	65.7
Age (years)		
6–10	73	19
11–15	312	81
Residence		
Rural	97	25.2
Urban	288	74.8
Mothers/guardian educational status		
Illiterate	144	37.4
Read and write	153	39.7
Primary school	62	16.1
Secondary school and above	26	6.8
Respondent grade		
One	46	11.9
Two	30	7.8
Three	27	7.0
Four	25	6.5
Five	47	12.2
Six	69	17.9
Seven	64	16.6
Eight	77	20.0

Total	385	100

**Table 2:** Prevalence of *S.  mansoni* and common intestinal helminths by sex among school children in Sanja General Elementary School, Sanja, northwest Ethiopia, 2013.

Parasite	Male	Female	Total	*P* value	*χ* ^2^
	*N* = 132	*N* = 253	*N* = 385		
*S. mansoni *	119 (34.4)**	227 (65.6)	346 (89.9)	0.895	0.02
Hookworm	49 (34.3)	94 (65.7)	143 (37.1)	0.995	0.00
*A. lumbricoides *	24 (38.7)	38 (61.3)	62 (16.1)	0.423	0.46
*T. trichiura *	3 (100)	0 (0)	3 (0.8)	0.016*	5.80
*Taenia* species	0 (0)	6 (100)	6 (1.6)	0.075	3.18
*H. nana *	3 (41.9)	18 (58.1)	31 (8.1)	0.47	3.94

Total	124 (33.3)	248 (66.7)	372 (96.6)	0.035*	4.44

*The difference was statistically significant (*P* < 0.05).

**Figures in parenthesis indicate percentages.

**Table 3:** The intensity of *Schistosoma mansoni* and common intestinal helminths using Kato-thick smear technique among Sanja General Elementary School children, northwest Ethiopia, 2013.

Parasite	Intensity		
	Light	Moderate	Heavy
*S. mansoni *	71 (18.4)**	181 (47)	72 (18.7)
*A. lumbricoides *	25 (6.5)	0 (0)	0 (0)
Hookworm	29 (7.5)	0 (0)	0 (0)
*T. trichiura *	1 (0.3)	0 (0)	0 (0)

Total	126 (32.7)	181 (47)	72 (18.7)

**Figures in parenthesis indicate percentages.

**Table 4:** Multivariate analysis for factors potentially associated with *Schistosoma  mansoni* infection among Sanja General Elementary School children, northwest Ethiopia, 2013.

Risk factors	*Schistosoma mansoni* number (%)			OR (95% CI)		*P* value
	Positive	Negative	Total	COR	AOR	
Sex						
Male	119 (90.2)	13 (9.8)	132 (34.3)	0.95 (0.45, 2.02)	0.86 (0.41, 1.80)	0.69
Female	227 (89.7)	26 (10.3)	253 (65.7)	100	100	
Age (years)						
6–10	66 (90.4)	7 (9.6)	73 (19.0)	0.93 (0.36, 2.32)	0.63 (0.18, 2.20)	0.47
11–15	280 (89.7)	32 (10.3)	312 (81.0)	100	100	
Resident						
Rural	86 (88.7)	11 (11.3)	97 (25.2)	100	100	0.53
Urban	260 (90.3)	28 (9.7)	288 (74.8)	0.84 (0.38, 1.89)	0.78 (0.37, 1.66)	
Grade						
One–four	118 (92.2)	10 (7.8)	128 (33.2)	0.67 (0.29, 1.49)	2.02 (0.67, 6.10)	0.22
Five–eight	228 (88.7)	29 (11.3)	257 (66.8)	100	100	
Mothers/guardian edu. status						
Illiterate	131 (91.0)	13 (9.0)	144 (37.4)	0.50 (0.20, 1.26)	0.43 (0.13, 1.21)	0.36
Read and write/1ry	154 (91.7)	14 (8.3)	168 (43.6)	0.46 (0.19, 1.14)	0.37 (0.11, 1.09)	
2ry school and above	61 (83.6)	12 (16.4)	73 (19.0)	100	100	
Swimming in river						
Yes	106 (79.7)	27 (20.3)	133 (34.5)	5.09 (2.37, 11.1)	5.12 (3.47, 17.89)^•^	0.01
No	240 (95.2)	12 (4.8)	252 (65.5)	100	100	
Swimming frequency/wk						
0	70 (79.5)	18 (20.5)	88 (22.9)	1.00	1.00	0.00
1-2	170 (90.9)	17 (9.1)	187 (48.6)	2.57 (1.18, 5.60)	1.82 (0.79, 4.23)	
≥3	106 (96.4)	4 (3.6)	110 (28.5)	6.81 (2.05, 24.95)	4.83 (1.37, 18.81)^•^	
Washing clothes in river						
Yes	101 (79.5)	26 (20.5)	127 (33.0)	4.85 (2.28, 10.43)	5.54 (2.89, 13.76)^•^	0.00
No	245 (95.0)	13 (5.0)	258 (67.0)	100	100	
Washing utensil in river						
Yes	68 (78.2)	19 (21.8)	87 (22.6)	3.88 (1.86, 8.09)	4.91 (2.43, 12.37)^•^	0.00
No	278 (93.3)	20 (6.7)	298 (77.4)	100	100	
Crossing river with bare foot						
Yes	75 (77.3)	22 (22.7)	97 (25.2)	4.62 (2.25, 9.77)	3.95 (2.13, 7.61)^•^	0.00
No	271 (94.1)	17 (5.9)	288 (74.8)	100	100	
Fishing in the river						
Yes	110 (82.1)	24 (17.9)	134 (34.8)	3.20 (1.56, 6.62)	3.04 (2.14, 8.71)^•^	0.00
No	235 (93.6)	16 (6.4)	251 (65.2)	100	100	

Total	346 (89.9)	39 (10.1)	385 (100)			

^•^The difference was statistically significant (*P* < 0.05).
